# Is There a Role for SGLT2 Inhibitors in Patients with End-Stage Kidney Disease?

**DOI:** 10.1007/s11906-024-01314-3

**Published:** 2024-06-24

**Authors:** Rehma Siddiqui, Yoshitsugu Obi, Neville R. Dossabhoy, Tariq Shafi

**Affiliations:** 1 Division of Nephrology, Department of Medicine, University of Mississippi Medical Center, 2500 North State Street, Jackson, MS USA; 2 Division of Kidney Diseases, Hypertension, & Transplantation, Houston Methodist Hospital, Houston, TX USA

**Keywords:** Sodium-glucose cotransporter-2 inhibitors, End-stage kidney disease, Dialysis, Residual kidney function, Chronic kidney disease, Heart failure, Mortality, Oxydative stress, Autophagy, Inflammation

## Abstract

**Purpose of Review:**

Chronic kidney disease and end-stage kidney disease (ESKD) are well-established risk factors for cardiovascular disease (CVD), the leading cause of mortality in the dialysis population. Conventional therapies, such as statins, blood pressure control, and renin-angiotensin-aldosterone system blockade, have inadequately addressed this cardiovascular risk, highlighting the unmet need for effective treatment strategies. Sodium–glucose transporter 2 (SGLT2) inhibitors have demonstrated significant renal and cardiovascular benefits among patients with type 2 diabetes, heart failure, or CKD at risk of progression. Unfortunately, efficacy data in dialysis patients is lacking as ESKD was an exclusion criterion for all major clinical trials of SGLT2 inhibitors. This review explores the potential of SGLT2 inhibitors in improving cardiovascular outcomes among patients with ESKD, focusing on their direct cardiac effects.

**Recent Findings:**

Recent clinical and preclinical studies have shown promising data for the application of SGLT2 inhibitors to the dialysis population. SGLT2 inhibitors may provide cardiovascular benefits to dialysis patients, not only indirectly by preserving the remaining kidney function and improving anemia but also directly by lowering intracellular sodium and calcium levels, reducing inflammation, regulating autophagy, and alleviating oxidative stress and endoplasmic reticulum stress within cardiomyocytes and endothelial cells.

**Summary:**

This review examines the current clinical evidence and experimental data supporting the use of SGLT2 inhibitors, discusses its potential safety concerns, and outlines ongoing clinical trials in the dialysis population. Further research is needed to evaluate the safety and effectiveness of SGLT2 inhibitor use among patients with ESKD.

## Introduction

Chronic kidney disease (CKD) and end-stage kidney disease (ESKD) are associated with an increased risk of CVD and mortality. CKD has the bidirectional relationship with cardiovascular disease (CVD). The manifestations of CVD in CKD can be broadly classified as myocardial remodeling (i.e., left ventricular hypertrophy, systolic and diastolic dysfunction) and vascular remodeling (i.e., atherosclerosis, arteriosclerosis, vascular calcification), which interact with each other [1]. CVD is the leading cause of mortality in the dialysis population, accounting for 45% of all deaths. The prevalence of coronary heart disease, heart failure, and left ventricular hypertrophy is reported as high as 40%, 43%, and 70%, respectively [2, 3]. Particularly, heart failure poses a significant challenge in the management of ESKD. It frequently develops after initiation of dialysis and is a prominent mortality risk factor among these patients [4]. Traditional therapies to prevent CVD complications in the general population have shown to be ineffective in CKD. To address the unmet need, further research is needed to evaluate novel therapeutic strategies to improve cardiovascular outcomes among patients on dialysis.

Sodium-glucose transporter type 2 (SGLT2) inhibitors have been shown to confer substantial kidney and cardiovascular benefits among patients with type 2 diabetes, heart failure, and/or high-risk CKD [5, 6]. In the current clinical landscape, SGLT2 inhibitors can be initiated among patients with eGFR 20 ml/min/1.73 m^2^ or more but need to be discontinued upon dialysis initiation for EKSD [7]. This practice likely stems from the diminished efficacy of SGLT2 inhibitors in promoting glucosuria and natriuresis as kidney function declines [8, 9], coupled with undetermined safety profiles among patients with advanced CKD or ESKD who were historically excluded from pivotal clinical trials. Nevertheless, recent emerging data indicate that SGLT2 inhibitors may provide cardiovascular benefits even among dialysis-dependent patients with low or absent kidney function. This evolving perspective may gain momentum now that the Food and Drug Administration (FDA) has removed dialysis dependency from the list of contraindications in the drug labels of SGLT2 inhibitors in 2023, after a multi-discipline review acknowledging the current data and evolution in the understanding of this class of agents [10, 11].

This article aims to comprehensively review the hypothesized clinical advantages, their postulated pathways, and potential safety issues associated with the use of SGLT2 inhibitors in ESKD, with a special focus on heart failure. Additionally, we outline ongoing clinical trials of this drug class in the dialysis population.

### Current Clinical Evidence of SGLT2 Inhibitors among Non-Dialysis Patients

Sodium-glucose transporter type 2 (SGLT2) inhibitors are initially approved by the FDA for the management of type 2 diabetes. SGLT2 is mainly expressed in the kidneys, specifically in the apical membrane of the S1 and S2 segments of the proximal tubule. SGLT2 inhibitors induce glycosuria and natriuresis by inhibiting sodium and glucose reabsorption, leading to improved glycemic control, small reduction in blood pressure, and mild to modest weight loss among patients with type 2 diabetes [12, 13]. Interestingly, unlike traditional diuretics, SGLT2 inhibitor-induced diuresis is associated with fewer electrolyte abnormalities, a decreased risk of acute kidney injury, and less neurohormonal activation [8]. Additionally, SGLT2 inhibitor use lowers the risk of hyperkalemia [14], thereby facilitating the continuation of the combined regimen with renin-angiotensin-aldosterone system inhibitors [15]. Beyond the renal effects of glycosuria and natriuresis, SGLT2 inhibitors also offer broad metabolic benefits, including reduction in visceral, liver, and epicardial fat by shifting substrate utilization from carbohydrates to lipids and ketone bodies [16–20], decreased serum uric acid levels and lowered risk of gout flares via enhanced renal uric acid excretion [21, 22], reduced kidney stone formation by increasing urine citrate levels [23–26], and alleviation of anemia by increasing erythropoietin production and suppressing proinflammatory pathways [27]. Cardiovascular outcome trials revealed that SGLT2 inhibition among patients with type 2 diabetes reduced the risk of cardiovascular events, cardiovascular mortality, and all-cause mortality, with consistency of favorable heart failure and kidney outcomes across the drug class [5, 6].

It should be noted that SGLT2 inhibitors are the first drug class that has shown clear efficacy on clinical hard endpoints in both heart failure with reduced ejection fraction (HFrEF) and preserved ejection fraction (HFpEF). Furthermore, recent clinical trials have shown promising cardiac benefits of the drug regardless of diabetes status [28–34]. Additionally, recent meta-analyses of clinical trials showed that SGLT2 inhibitor use is associated with a lower risk of atrial fibrillation/flutter events [35, 36]. Observational studies suggest that SGL2 inhibitors may stabilize atherosclerotic plaque among patients with type 2 diabetes and ischemic heart disease [37, 38], thereby reducing major adverse cardiovascular events [39, 40], but meta-analyses showed their neutral effects on stroke or myocardial infarction [41, 42].

While SGLT inhibitors lead to improved glycemic control, better blood pressure management, and weight reduction, these factors alone do not fully explain the extensive cardiac benefits conferred by this drug class. Given the close relationship between the severity of CKD and the increased risk of CVD, the renoprotective effect of SGLT2 inhibitors is considered to play a significant role. This perspective is supported by multiple clinical trials demonstrating reduced albuminuria and a lowered risk of CKD progression by SLGT2 inhibition, benefits that are evident in both diabetic and non-diabetic patient populations [7, 43].

### Potential Cardiovascular Benefits of SGLT2 Inhibitors among Dialysis Patients

**Fig. 1:**
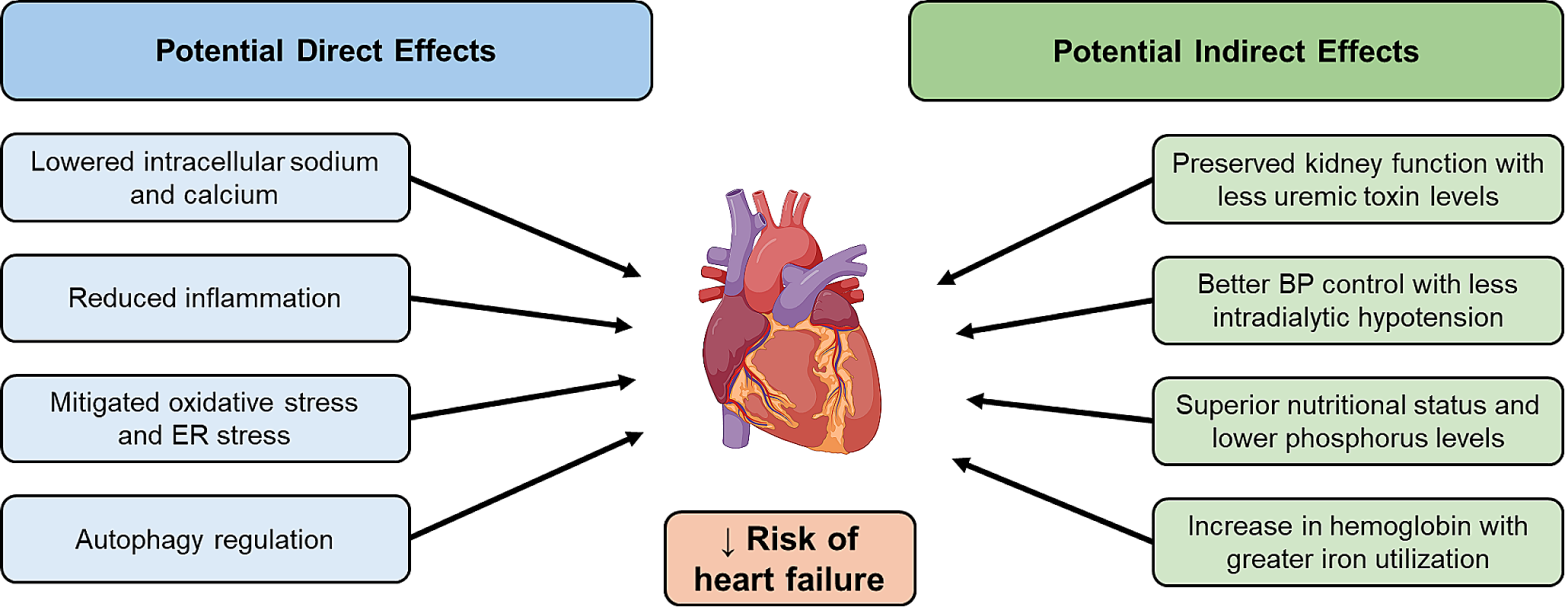
Potential direct and indirect cardiovascular benefit of SGLT2 inhibitors in the dialysis population. Potential indirect benefits include preservation of kidney function, which can lead to multiple benefits from various aspects including uremic toxin levels, volume and blood pressure (BP) control, and nutrition. SGLT2 inhibitors also improve anemia and iron utilization, leading to less requirement of erythropoietin stimulating agents (ESAs), which is known to increase the risk of cardiovascular events. SGLT2 inhibitors also directly act on cardiomyocytes and endothelial cells and lower intracellular sodium and calcium level, reduce inflammation, mitigate oxidative stress and endoplasmic reticulum (ER) stress, and regulate autophagy. Created using BioRender.com

Urinary glucose excretion induced by SGLT2 inhibitors linearly diminishes with lower kidney function [44], and their plasma glucose-lowering effect is attenuated in patients with eGFR < 60 ml/min per 1.73 m2 and becomes negligible when eGFR is < 30 ml/min per 1.73 m2 [45]. Nevertheless, the benefits of SGLT2 inhibitors in kidney and cardiovascular outcomes are generally preserved among patients with CKD and are observed even among non-diabetic patients. For instance, the DAPA-CKD trial showed that dapagliflozin reduced the risk of kidney, cardiovascular and mortality endpoints even in a subgroup of patients with stage 4 CKD, which was consistent with those observed in the entire study [29, 46]. The EMPA-KIDNEY trial also demonstrated that empagliflozin significantly reduced the risk of CKD progression or cardiovascular death across eGFR levels extending to stage 4 CKD [47]. Such kidney function-independent benefits were confirmed in a recent meta-analysis of over 90,000 participants from randomized, placebo-controlled clinical trials [48]. Interestingly, the most substantial risk reduction for heart failure outcomes was observed among patients with lower eGFR levels. Collectively, these findings warrant clinical studies evaluating the effects of SGLT2 inhibitors on cardiovascular outcomes among dialysis patients, where these drugs may provide indirect and direct cardiovascular benefits (Fig. 1).

### Indirect Cardiovascular Benefits of SGLT2 Inhibitors

Recent clinical studies have shown that residual kidney function, even at such low levels observed among dialysis patients, is closely associated with better clinical outcomes. In addition to better clearance of uremic toxins, greater residual kidney function is associated with less inflammation, better quality of life, fewer episodes of intradialytic hypotension, better nutritional status, more effective control of phosphorus, less pill burden, less requirement of erythropoietin stimulating agents (ESAs), a lower risk of cardiovascular events, and a lower risk of death [49–54]. . The majority of incident ESKD patients still have some kidney function, with approximately 27% and 10% starting dialysis with an eGFR of 10–14 ml/min/1.73 m^2^ and ≥ 15 ml/min/1.73 m^2^, respectively [4]. SGLT2 inhibitors slows the progression of CKD, and hence, may help preserve residual kidney function even after dialysis initiation. This in turn could lower the risk of CVD including heart failure.

An additional indirect cardiovascular benefit of SGLT2 inhibitors is the mitigation of anemia and the improvement in iron utilization. Clinical trials have shown that these drugs can increase hematocrit levels by 1.9–2.4%, reduce the risk of developing anemia, and decrease the likelihood of needing iron supplements or ESA treatment [27, 55–59]. These effects are attributed to stimulation of ESA production and reduced hepcidin production by decreasing inflammation and activating nutrient deprivation signaling such as sirtuin-1 in the liver [60]. Notably, this benefit on anemia was consistently observed in moderate-to-severe CKD, where kidney erythropoietin production is impaired [55]. It is postulated that the activation of sirtuin-1 activation by SGLT2 inhibitors could stimulate hypoxia-inducible factor (HIF)-2α, leading to erythropoietin production in the liver [61]. Furthermore, unlike HIF prolyl hydroxylase inhibitors (HIF-PHI), experimental studies have shown that SGLT2 inhibitors suppressed the expression and activity of HIF-1α [62–66], albeit with some exceptions [67, 68]. This distinction could be an important property of this drug class because while HIF-1α does not significantly contribute to endogenous erythropoietin synthesis, it may enhance atherosclerotic plaque instability and promote cardiac fibrosis [69]. Additionally, HIF-2α stimulation by SGLT2 inhibitor use has been also reported in in vitro studies [70–72], indicating this effect is partly independent of glucosuria. Given the increased cardiovascular risk associated with ESA therapy with or HIF-PHI inhibitor [73], and considering the high prevalence and associated mortality risk of functional iron deficiency [74], SGLT2 inhibitors could become an attractive alternative in the management of anemia in the dialysis population.

### Direct Cardiovascular Benefits of SGLT2 Inhibitors

**Fig. 2:**
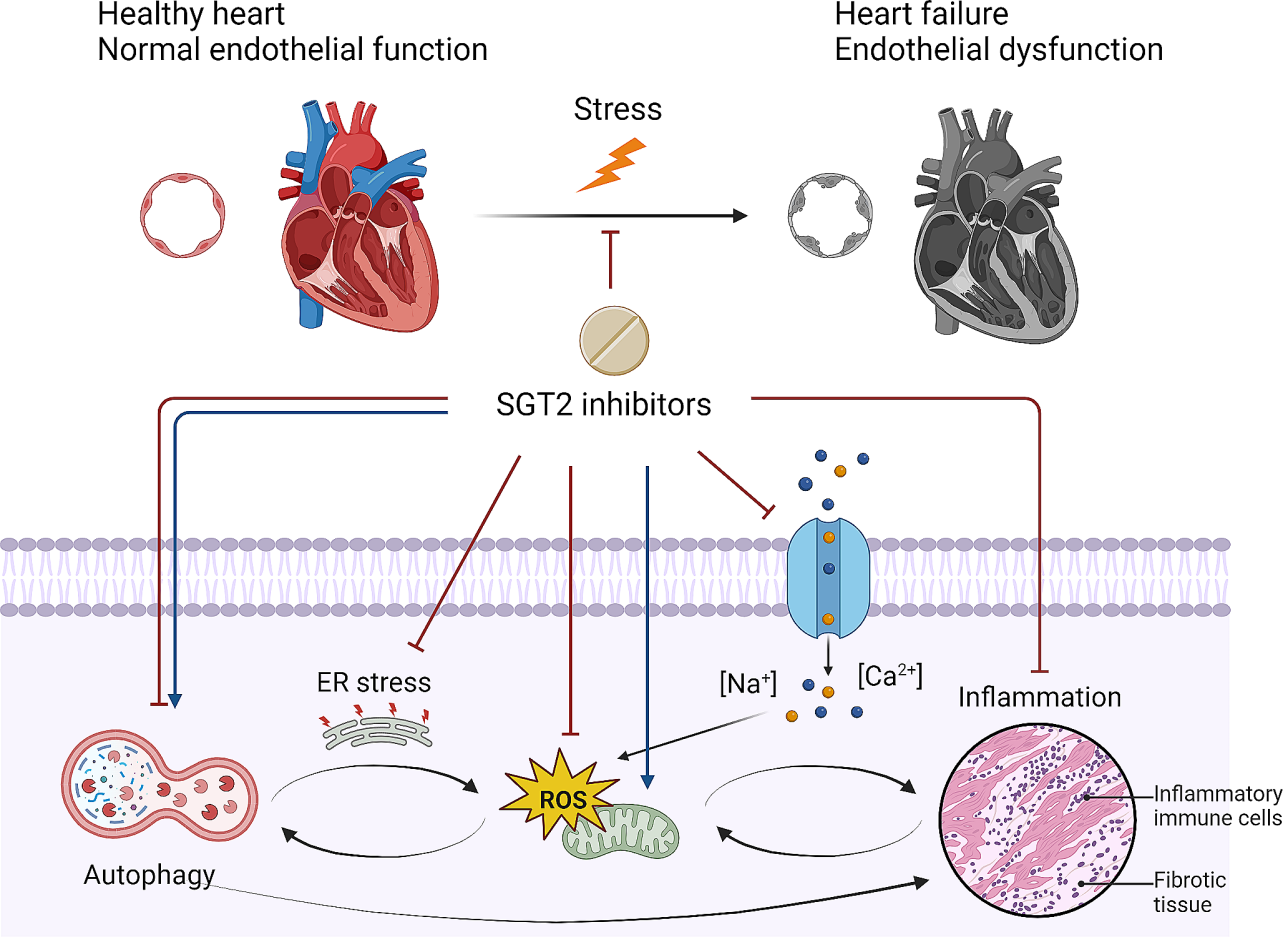
Potential pathways of direct cardiovascular benefits via SGLT2 inhibitor use. SGLT2 inhibitors can prevent or improve cardiac dysfunction and remodeling by lowering intracellular sodium and calcium levels, reducing oxidative and endoplasmic reticulum (ER) stress, suppressing the inflammatory process, and regulating autophagy. These mechanisms are interrelated. Created using BioRender.com

Recent preclinical and translational research has provided data supporting direct benefits of SGLT2 inhibitors on the cardiovascular system, including cardiomyocytes, endothelial cells, and smooth muscle cells. Experimental studies using ex vivo isolated perfused hearts showed that empagliflozin mitigated ischemia-reperfusion injury and improved cardiac output, contractile dysfunction, and ventricular arrhythmia vulnerability [75, 76]. Given the minimal SGLT2 expression in the heart [77, 78], extensive research has been conducted to identify the mechanisms behind the direct cardiovascular effects of SGLT2 inhibitors. First, several clinical studies revealed increased SGLT2 expression in endomyocardial biopsy samples from patients with various heart conditions [79–81]. Second, molecular docking analysis indicated that empagliflozin could bind with other glucose transporters (i.e., facilitated-diffusion glucose transporters [GLUT], SGLT1, and NHE) with much higher affinity for GLUT1 and GLUT4 compared with SGLT1 and NHE [76]. It should also be noted that SGLT2 inhibitors have variable selectivity for SGLT2 vs. SGLT1, i.e., 2500× selectivity for empagliflozin, 1200× selectivity for dapagliflozin, 250× selectivity for canagliflozin, and 20x selectivity for sotagliflozin [82], and less selective SGLT2 inhibitors was associated with a lower risk of heart failure in network meta-analyses [83, 84]. Third, in vitro studies using cardiomyocytes have shown that SGLT2 inhibitors elicit cellular responses without glucose in the medium [85], suggesting glucose transporter-independent mechanisms. The exact pathways of the direct cardioprotective effects of SGLT2 inhibitors remain to be fully elucidated but appear to involve various processes in the pathophysiology of heart failure, such as regulation of intracellular electrolytes, inflammation, oxidative stress, mitochondrial function, and autophagic flux [24, 86] (Fig. 2).

1. Intracellular electrolyte regulation.

Increases in myocardial intracellular sodium and calcium levels, coupled with a subsequent decrease in mitochondrial calcium levels, are recognized as early hallmarks and contributors of cardiovascular death and heart failure [86, 87]. Several preclinical studies have shown that SGLT2 inhibitors can counteract these adverse changes by directly inhibiting sodium-hydrogen exchanger 1 (NHE-1) in cardiomyocytes and endothelial cells [85–88]. This inhibition leads to reduced intracellular sodium levels, followed by a decrease in intracellular calcium levels alongside an increase in mitochondrial calcium levels, likely through the action of sodium-calcium exchangers. SGLT2 inhibitors also inhibit hydrogen peroxide-induced late sodium current in cardiomyocytes with little effect on peak sodium current, which may protect against arrhythmias associated with prolonged action potentials [75, 89].

Of note, a phase III randomized, placebo-controlled clinical trial evaluated the effect of NHE-1 inhibition by cariporide among 5761 patients undergoing high-risk coronary artery bypass graft surgery and found that cariporide significantly reduced the incidence of myocardial infarction but increased mortality from cerebrovascular events [90]. SGLT2 inhibitors may have an advantage on this regard because their use has been shown to have neutral effect on overall cerebrovascular events [41, 42]. However, the direct NHE-1 inhibition by SGLT2 inhibitors were not consistently observed in other studies [91–93], indicating the presence of unknown effect modifying factors.

2. Oxidative stress and inflammation.

Oxidative stress and inflammation are interdependently involved in the pathogenesis of CVD, perpetuating a chronic and vicious cycle with heart failure. Oxidative stress, caused by reactive oxygen species (ROS), induces the inflammation pathway through the activation of nucleotide-binding domain, Leucine-rich-containing family, pyrin domain-containing-3 (NLRP3) inflammasome [94]. The subsequent release of inflammatory cytokines, if excessive, can lead to inflammatory cell death, known as pyroptosis [95]. In turn, inflammation also induces oxidative stress via various cellular signaling pathways involving mediators such as protein kinase C and calcium. These mediators activate sources of reactive oxygen species (ROS) such as nicotinamide adenine dinucleotide phosphate (NADPH) oxidase and the mitochondrial electron transport chain. Chronic inflammation and oxidative stress promote proinflammatory macrophage infiltration and augment interstitial collagen deposition, which creates areas of replacement fibrosis, eventually leading to progressive left ventricular remodeling and dysfunction [96, 97]. ROS reduces nitric oxide-cyclic guanosine monophosphate-protein kinase G (NO-cGMP-PKG) signaling, leading to myocardial hypertrophy and increased stiffness by diminishing myofilament phosphorylation [98]. Proinflammatory macrophage infiltration and decreased NO production in the endothelial cells play a significant role in the progression of atherosclerosis [99].

In the myocardium of HFrEF, myocardial injury or death is triggered by oxidative stress from various stimuli such as ischemia, pressure overload, or toxicity, followed by inflammatory responses. Activation of sympathetic nervous system and renin-angiotensin-aldosterone system can also induce sustained myocardial inflammation [100]. Conversely, among patients with HFpEF, comorbid conditions such as aging, overweight/obesity, diabetes, sleep apnea, atherosclerotic disease, and smoking/chronic obstructive pulmonary disease are commonly present and known to cause chronic and systemic inflammation, which then induces oxidative stress [96, 97].

SGLT2 inhibitors have been shown to reduce the expression of various circulating inflammatory molecules (e.g., Interleukin [IL]-1β, IL-6, and IL-18, tumor necrosis factor-α [TNF-α], monocyte chemoattractant protein-1 [MCP-1]) and cell adhesion molecules across different studies [101, 102]. Potential mechanisms underlying the anti-inflammatory effects of SGLT2 inhibitors include the reduction in intracellular calcium levels via NHE-1 inhibition and activation of 5’ adenosine monophosphate-activated protein kinase (AMPK), both leading to the suppression of the NLRP3 inflammasome activation [103]. In murine models of doxorubicin-induced cardiomyopathy, empagliflozin reduced ferroptpsis, fibrosis, apoptosis, and inflammation through the involvement of NLRP3 and myddosome-related pathways, leading to improved cardiac functions [104]. Additionally, empagliflozin was shown to suppress the increase in the expression of proinflammatory makers induced by doxorubicin in vitro.

The cardiovascular benefits of SGLT2 inhibitors are further seen through oxidative stress reduction. For instance, empagliflozin attenuated cardiomyocyte hypertrophy, diminished interstitial fibrosis, and reduced myocardial oxidative stress in non-diabetic rats with left ventricular dysfunction post-myocardial infarction [105]. Furthermore, in an in vitro study, empagliflozin restored the endothelium-mediated cardiomyocyte relaxation and contraction, which was impaired due to decreased nitric oxide availability and increased mitochondrial ROS following exposure to uremic serum from patients with ESKD [106]. Such anti-inflammatory and anti-oxidative properties of SGLT2 inhibitors can also lead to improvements in endothelial function and arterial wall stiffness as shown in both animal and clinical models [92, 107, 108] and may mitigate the development of cardiac fibrosis and atherosclerosis by inhibiting macrophage infiltration, reducing foam cell formation, and promoting macrophage polarization from pro-inflammatory M1 subtype to anti-inflammatory M2 subtype [38, 101, 109, 110].

3. Autophagy Regulation.

Autophagy-lysosome pathway is primarily a catabolic process that maintains cellular homeostasis. It captures misfolded proteins, damaged organelles, and pathogens in autophagosomes for degradation by lysosomal proteases [111]. This process plays an important role in facilitating metabolic adaptation, preventing cellular damage, and preserving genomic stability. This catabolic process is activated in response to various stressors—such as shear stress, hypoxia, ischemia, and mitochondrial damage—via crucial signaling networks such as mTOR, AMPK, glycogen synthase kinase 3 beta (GSK-3β), and the Hippo pathway [112]. Impaired autophagy can contribute to the accumulation of cellular debris, dysfunctional mitochondria, and NLRP3 inflammasome activators and components, leading to cellular stress and inflammation [113]. Autophagy is particularly important for cardiomyocytes, the terminally differentiated cells that infrequently undergo cell division.

While essential for cardiac function and limiting disease progression post-injury, an imbalance in autophagy levels—either suppression or excessive activation—can lead to or exacerbate pathological outcomes [114]. For instance, autophagy plays an adaptive role in progressive heart failure and protects myocardial cells, and autophagy activity was associated with left ventricular reverse remodeling among patients with dilated cardiomyopathy [80]. In the late period of heart failure, however, substances and injury myocardial cells can be overly removed via autophagic pathway. Excessive autophagy activation may also occur with pressure overload or ischemia/reperfusion injury [115–117]. Therefore, maintaining an “optimal window” of autophagy activity according to disease conditions is crucial for cellular homeostasis [114].

SGLT2 inhibitors have been shown to “modulate” cardiac autophagy and lysosomal degradation. These drugs promote autophagy through glucosuria-induced upregulation of nutrient deprivation signals such as AMPK, sirtuins, and peroxisome proliferator–activated receptor-γ coactivator (PGC)-1α, while downregulating nutrient surplus signals, including mTOR [118]. Consequently, this contributes to the improvement of mitochondrial morphology, function, and biogenesis in the heart, as shown in several in vivo studies [119, 120]. Another in vivo study showed that empagliflozin ameliorated sunitinib-induced cardiac dysfunction by restoring AMPK-mTOR mediated autophagy in mice [121]. Conversely, Jiang, et al. showed that empagliflozin reduced cardiac infarct size and fibrosis and resulted in improved cardiac function and survival in mouse models and reported that those cardioprotective effects were at least in part through downregulation, not upregulation, of excessive autophagic flux through NHE-1 inhibition [122]. Empagliflozin also inhibited hyperactivation of autophagy in murine diabetic cardiomyopathy by inhibiting GSK-3β, resulting in reversal of cardiac dysfunction [123]. Importantly, such autophagy upregulation via AMPK activation and downregulation via NHE-1 or GSK-3β inhibition were also confirmed in accompanying in vitro studies [121–123]. Those findings indicate that the anti-inflammatory and anti-oxidative effects on SGLT2 inhibitors are at least partly independent of their effects on the kidneys (i.e., glucosuria and natriuresis), suggesting a potential role in ESKD.

4. Endoplasmic reticulum stress.

Endoplasmic reticulum (ER) is a critical cellular organelle involved in protein folding and secretion, calcium storage, and lipid and carbohydrate metabolism [124]. ER stress is a response to proteostasis imbalance such as the accumulation of misfolded or unfolded proteins. ER stress-induced inflammation can help limit tissue damage and promote tissue repair; however, the effects of ER stress-induced inflammation depend on the type of ER stressor, the disease stage, and the target cell type [125]. Oxidative stress also occurs alongside ER stress as the misfolded proteins produce ROS during attempts to refold, which disturbs cellular redox balance. This oxidative stress can further exacerbate ER stress, creating a vicious cycle that can lead to cell damage and disease [125, 126]. The disruption in ER homeostasis intricately activates the unfolded protein response (UPR) and autophagy to restore normal function by halting protein translation, degrading misfolded proteins, and activating the signaling pathways that increase the production of molecular chaperones [124, 127].

Recent studies have shown that SGLT2 inhibitors have protective effects against ER stress in cardiomyocytes. Treatment with SGLT2 inhibitors has been shown to reduce the expression of key ER stress markers such as cleaved caspase 3, Bax, activating transcription factor 4, C/EBP homologous protein, and glucose-regulated protein78 in cardiomyocytes exposed to high glucose [128], hydrogen peroxide [129], angiotensin II [130], or doxorubicin [131, 132] in both in vivo and in vitro studies. These findings suggest that SGLT2 inhibitors may directly prevent the initiation of cell death pathways triggered by ER stress in the heart.

### Safety Considerations in the Use of SGLT2i in ESKD

Previous pharmacokinetic studies showed that among patients with advanced CKD and ESRD, when compared to those with normal kidney function, a single-dose administration generally resulted in similar peak plasma levels, a mildly prolonged half-life time, and approximately 1.5-times larger AUC [133]. There appeared to be no clinically meaningful difference in those pharmacokinetic parameters from stage 4 CKD through ESRD. Additionally, 7 days of dapagliflozin 10 mg/day among dialysis patients resulted in no significant drug accumulation but peak concentrations similar to those observed among the age- and sex-matched control patients with normal kidney function [134].

From a clinical safety standpoint, the EMPA-REG Renal trial showed the risk of mild to moderate urinary tract infection associated with empagliflozin use was more pronounced among patients with more advanced CKD (i.e., 18.9% in the empagliflozin group vs. 8.1% in the placebo group in stage 4 CKD; no acute pyelonephritis or urosepsis was reported) [135]. This warrants caution in the use of SGLT2 inhibitors among oliguric dialysis patients. However, in the DAPA-CKD trial, dapagliflozin did not show increased risk of adverse events across subgroups including CKD stage 4, despite continuation of dapagliflozin even when eGFR declined to < 15 ml/min per 1.73m^2^ [46].

## Ongoing Clinical Trials

Several clinical trials are underway to investigate the effects of SGLT2 inhibitors in the dialysis population (Table 1). Such trials registered in CliniclTrials.gov include RENAL LIFECYCLES (NCT05374291), DAPA-HD (NCT05179668), and SIP-AkiD (NCT05309785). RENAL LIFECYCLES aims to enroll 1500 patients with either advanced CKD (eGFR < 25 ml/min per 1.73 m2), ESKD requiring dialysis with residual diuresis > 500 ml/day, or transplant kidney allograft with eGFR < 25 ml/min per 1.73 m2 and to evaluate a composite endpoint of hard clinical outcomes, i.e., all-cause mortality, kidney failure, and hospitalization for heart failure. Many other studies have cardiac imaging parameters or brain natriuretic peptide levels as the primary outcomes. New trials include CANARY (NCT05715814), CARe-MRI (NCT06182839), SEED (NCT05786443), EMPA-PRED (NCT06249945), and EMPA-RRED (NCT06249932) which are expected to begin early 2024. The results of these trials are expected to provide proof-of-concept evidence on the efficacy and safety of SGLT2 inhibitors, a potentially important step towards the better management of cardiovascular disease among dialysis patients.

**Table 1:** Summary of ongoing clinical trials evaluating the effects of SGLT2 inhibitors in the dialysis population registered in ClinicalTrials.gov as of April 6, 2024

NCT Number	Interventions	Outcome Measures	Target *N*	Randomization	Placebo	Start Date	Completion Date
NCT06249945	Empagliflozin	Echocardiogram parameters	150	Yes - Parallel	Yes	2/1/2024	12/31/2030
NCT06249932	Empagliflozin	Cardiac MRI parameters	95	Yes - Parallel	Yes	2/1/2024	12/31/2030
NCT06182839	Canagliflozin	Cardiac MRI parameters	92	Yes - Parallel	Yes	3/30/2024	3/30/2029
NCT05967156	Empagliflozin	BNP	15	No - Single arm	No	6/1/2023	3/1/2024
NCT05965440	Dapagliflozin	Intestinal microbiota	50	No - Single arm	No	10/2/2023	12/15/2024
NCT05786443	Empagliflozin	Body fluid distributions	60	Yes - Parallel	Yes	1/31/2024	12/30/2025
NCT05737186	SGLT2 inhibitor	Quality of Life	40	Yes - Parallel	No	3/9/2023	12/31/2024
NCT05715814	Empagliflozin	Measured GFR	20	No - Single arm	No	2/1/2024	4/1/2025
NCT05687058	Empagliflozin	Feasibility	24	No - Parallel	No	11/1/2023	12/31/2024
NCT05685394	Dapagliflozin	NT-proBNP	80	Yes - Parallel	No	1/24/2023	12/1/2024
NCT05671991	Empagliflozin	Peritoneal glucose absorption	30	Yes - Crossover	Yes	3/1/2023	12/31/2024
NCT05614115	Empagliflozin	Feasibility	75	Yes - Sequential	Yes	3/21/2023	3/31/2025
NCT05374291	Dapagliflozin	All-cause mortality, kidney failure, and heart failure	1500	Yes - Parallel	Yes	11/8/2022	1/1/2027
NCT05309785	Canagliflozin	Pharmacokinetics	44	No - Single arm	No	11/24/2022	2/1/2025
NCT05179668	Dapagliflozin	Cardiac MRI parameters	108	Yes - Parallel	Yes	10/1/2022	9/30/2025

Abbreviations: SGLT2, sodium-glucose transporter-2; BNP, brain natriuretic peptide; MRI, magnetic resonance imaging

## Conclusions

The pleiotropic effects of SGLT2 inhibitors, including their benefits on preserving kidney function and improving cardiovascular health, make this drug class a promising therapeutic agent in the management of dialysis patients with ESKD. Clinical trials have demonstrated the cardiovascular benefits of SGLT2 inhibitors even among patients with advanced CKD, where its primary glycosuric effect is substantially diminished. Additionally, preclinical studies suggest potential direct actions of SGLT2 inhibitors in the cardiovascular system, where SGLT2 expression is minimal or negligible. These findings underscore the potential of SGLT2 inhibitors in preventing cardiovascular complications among dialysis patients. Ongoing pilot clinical trials are expected to provide preliminary results to evaluate whether larger clinical trials would be warranted from both safety and efficacy standpoint.

## Data Availability

No datasets were generated or analysed during the current study.
